# Discrete Fourier Transform Radar in the Terahertz-Wave Range Based on a Resonant-Tunneling-Diode Oscillator †

**DOI:** 10.3390/s21134367

**Published:** 2021-06-25

**Authors:** Hiroki Konno, Adrian Dobroiu, Safumi Suzuki, Masahiro Asada, Hiroshi Ito

**Affiliations:** 1 Tokyo Institute of Technology, Meguro, Tokyo 152-8550, Japan; konno.h.ac@m.titech.ac.jp (H.K.); suzuki.s.av@m.titech.ac.jp (S.S.); asada@pe.titech.ac.jp (M.A.); 2 Center for Natural Sciences, Kitasato University, Kanagawa 252-0373, Japan; h.ito@kitasato-u.ac.jp

**Keywords:** terahertz-wave radar, resonant-tunneling diode, optical coherence tomography, stepped-frequency continuous-wave radar

## Abstract

We used a resonant-tunneling-diode (RTD) oscillator as the source of a terahertz-wave radar based on the principle of the swept-source optical coherence tomography (SS-OCT). Unlike similar reports in the terahertz range, we apply the stepwise frequency modulation to a subcarrier obtained by amplitude modulation instead of tuning the terahertz carrier frequency. Additionally, we replace the usual optical interference with electrical mixing and, by using a quadrature mixer, we can discriminate between negative and positive optical path differences, which doubles the measurement range without increasing the measurement time. To measure the distance to multiple targets simultaneously, the terahertz wave is modulated in amplitude at a series of frequencies; the signal returning from the target is detected and homodyne mixed with the original modulation signal. A series of voltages is obtained; by Fourier transformation the distance to each target is retrieved. Experimental results on one and two targets are shown.

## 1. Introduction

The terahertz electromagnetic range, with frequencies roughly between 0.1 and 10 THz, has seen an increasing number of applications in imaging, spectroscopy, wireless communication, etc. Much of the interest for the terahertz radiation comes from its particular properties, mainly the ability to pass through various media that prevent or hinder the propagation of electromagnetic waves in other frequency bands—packaging materials (paper, cloth, plastics) and degraded visual environments (smoke, fog, dust) among others—while at the same time having a short enough wavelength to allow imaging or detection of small targets or features as well as propagation over long distances by using relatively small optics.

Terahertz-wave radars are among the applications benefiting from these properties. Initially, the positions and shapes of targets in the propagation direction were determined exclusively by using the time-domain spectroscopy, a time-of-flight technique whereby the arrival time of terahertz radiation pulses was used to measure propagation distances. Other ranging techniques now use the frequency-modulated continuous-wave (FMCW) radar principle [1,2,3,4], the amplitude-modulated continuous-wave (AMCW) radar principle [5,6], interferometry [7], or computed tomography [8]. In recent years, a new technique has been added to the arsenal of terahertz-wave ranging: the optical coherence tomography (OCT), either in its original form, the time-domain (TD) OCT, in which a low-coherence (wide-spectrum) light source is used [9], or the source-swept (SS) OCT, relying on a tunable monochromatic source [10,11,12]. This report is a contribution to the SS-OCT type of radar.

Compared with other reports on terahertz-wave ranging using SS-OCT, our approach features a number of novelty elements. First, we use a resonant tunneling-diode (RTD) oscillator as the terahertz-wave source and, in fact, all other electronic parts in the circuit are semiconductor-based, which brings about the potential of future miniaturization and integration. Second, instead of optical interference, we use a quadrature mixing technique that allows us to double the measurement range without an increase in the measurement time. Third, while the usual SS-OCT method relies on tuning the emission frequency of the source, we modulate the amplitude to obtain a subcarrier and then tune the frequency of that subcarrier; this can be advantageous when the source emission power is more easily modulated than the oscillation frequency, as is the case of an RTD source.

In optics, the SS-OCT has often been used in medical applications such as in ophthalmology. In the radar community, the same principle has been known as the stepped-frequency continuous-wave (SFCW) radar, first described in the 1970s as a time-domain reflectometer [13] and used in ground-penetrating radar sensing among other applications.

We chose to call this method the discrete Fourier transform (DFT) radar, because both the Fourier transform calculation and the discrete nature of the recordings are fundamental to this ranging technique, as will become clear in the following sections.

This article is a greatly expanded version of a conference poster contribution [14]. In addition to providing details on the theoretical derivation and the experiments, this article contains previously unpublished results and reports an improved performance.

## 2. Measurement Principle

### 2.1. Measurement Setup

**Figure 1:**
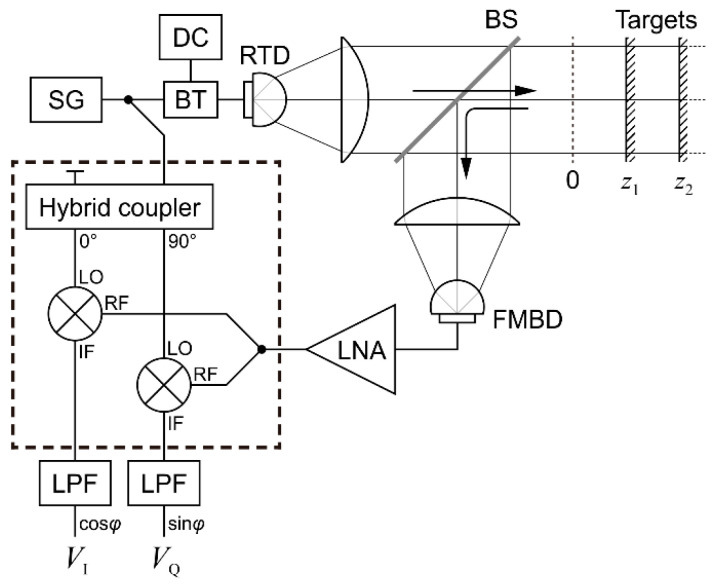
Schematic of the measurement setup. SG: signal generator; BT: bias tee; DC: power supply; BS: beam splitter; FMBD: Fermi-level-managed barrier diode detector; LNA: low-noise amplifier; LO: local oscillator input; RF: radio frequency input; IF intermediate frequency output; LPF: low-pass filter. Two power dividers are represented symbolically. The dashed rectangle is a commercial connectorized IQ mixer.

Figure 1 shows a schematic of the measurement system used in this study. The signal generator modulates the oscillation amplitude of the RTD source, from where the output terahertz wave is collimated and sent towards the targets. The reflected beam is extracted by a beamsplitter and sent to the detector, which demodulates the signal (removes the terahertz frequency carrier). An IQ mixer, containing a 90° phase shifter and two mixers, is fed with an LO signal from the same signal generator and with an RF signal from the detector. Since both LO and RF signals have the same frequency, the homodyne mixing produces DC signals at the two IF outputs; these are filtered, digitized, and processed.

The position 0 in the target area is where the propagation times through the reference arm (from SG to the mixers’ LO inputs) and the measurement arm (from SG to the RF inputs) are equal. The propagation occurs inside the various cables and devices as well as in air. The goal is to measure the positions of the targets along the optical propagation path.

### 2.2. Single Target

Let us first consider the simple case of a target consisting of a single reflecting surface. The modulated signal from the signal generator is

(1) $$V_{\mathrm{SG}}\left(t\right)=A_{\mathrm{SG}}\;\cos\left(2\pi ft\right),$$

where $A_{\mathrm{SG}}$ is the signal amplitude, f is the modulation frequency, and t is time.

After propagating through either arm, the reference signal $V_{\mathrm{ref}}$ arriving at the LO inputs and the measurement signal $V_{\mathrm{meas}}$ at the RF inputs are:

(2) $$V_{\mathrm{ref}}\left(t\right)=A_{\mathrm{ref}}\;\cos\left(2\pi f\left(t-t_{\mathrm{ref}}\right)\right),$$

(3) $$V_{\mathrm{meas}}\left(t\right)=A_{\mathrm{meas}}\;R\;\cos\left(2\pi f\left(t-t_{\mathrm{meas}}\right)\right),$$

where the amplitudes $A_{\mathrm{ref}}$ and $A_{\mathrm{meas}}$ now include gains and losses along each path and R is the reflectance of the target. The signals are delayed by the amounts $t_{\mathrm{ref}}$ and $t_{\mathrm{meas}}$, respectively.

The IQ mixer has two outputs, named I (from *in phase*) and Q (from *quadrature*). The signals obtained here are proportional to the product of the LO and RF signals, but the output Q is additionally shifted in phase by 90°.

First, the I output is the signal $V_{I}$:

(4) $$V_{I}\left(t\right)=V_{\mathrm{ref}}\left(t\right)\;V_{\mathrm{meas}}\left(t\right)\;g_{\mathrm{mix}}\;,$$

where $g_{\mathrm{mix}}$ is a coefficient accounting for the conversion gain of the mixer. Carrying out the multiplication gives the following result:

(5) $$V_{I}\left(t\right)=A_{\mathrm{ref}}\;A_{\mathrm{meas}}\;R\;g_{\mathrm{mix}}\cos\left(2\pi f\left(t-t_{\mathrm{ref}}\right)\right)\cos\left(2\pi f\left(t-t_{\mathrm{meas}}\right)\right),$$

(6) $$V_{I}\left(t\right)=A\;R\;\left[\cos\left(4\pi ft-2\pi ft_{\mathrm{ref}}-2\pi ft_{\mathrm{meas}}\right)+\cos\left(2\pi f\left(t_{\mathrm{meas}}-t_{\mathrm{ref}}\right)\right)\right],$$

with the resulting amplitude A. The first cosine in Equation (6) is an oscillation at double the modulation frequency, which is removed by low-pass filtering, such that finally, $V_{I}$ becomes a voltage that is constant in time and depends on the propagation time difference between the two arms and on the modulation frequency:

(7) $$V_{I}=A\;R\cos\left(2\pi f\left(t_{\mathrm{meas}}-t_{\mathrm{ref}}\right)\right).$$

The voltage at the Q output is calculated in the same way, but including the 90° phase shift, which gives:

(8) $$V_{Q}=A\;R\sin\left(2\pi f\left(t_{\mathrm{meas}}-t_{\mathrm{ref}}\right)\right).$$

Thus, two voltages in quadrature are obtained, from which the amplitude and the propagation time difference can be calculated as follows:

(9) $$A\;R=\sqrt{V_{I}{}^{2}+V_{Q}{}^{2}},$$

(10) $$t_{\mathrm{meas}}-t_{\mathrm{ref}}=\frac{1}{2\pi f}\mathrm{atan}2\left(V_{Q},V_{I}\right)+\frac{N}{f}\;,$$

where atan2 is the four-quadrant arc tangent function; it gives the angle whose sine and cosine are proportional to the two variables, respectively. The integer N is the unknown number of wavelengths comprised in the optical path difference.

If a single modulation frequency is used, this becomes an example of an AMCW radar. The target reflectance R can be obtained from the product A R after measuring the signal from a reference surface. The target distance can be calculated from $t_{\mathrm{meas}}-t_{\mathrm{ref}}$ by taking into account the speed of light in air. However, the unambiguous measurable range is determined by the range of values that the function atan2 can have, from −π to +π. This is very limiting, as the target distance can be allowed to vary only within half the wavelength corresponding to the modulation frequency; for example, with a modulation frequency of 5 GHz, half the wavelength is just 30 mm. To remove this limitation, our group proposed a technique that relies on switching from one modulation frequency to another, in a continuous manner, which allows the measurement of absolute distances in a virtually unlimited range [5,6].

If the modulation frequency is changed within a sufficiently wide interval and in sufficiently small steps, the values of $V_{I}$ and $V_{Q}$ will reconstitute two shifted sinusoids and the propagation time difference $t_{\mathrm{meas}}-t_{\mathrm{ref}}$ can be determined unambiguously.

### 2.3. Multiple Target

Let us now consider the case of a target consisting of two or more reflecting surfaces at different distances. In this case, the reference signal $V_{\mathrm{ref}}$ will remain unchanged, whereas the signal coming from the measurement arm will be the sum of the contributions from each reflecting surface:

(11) $$V_{\mathrm{meas}}\left(t\right)=A_{\mathrm{meas}}\overset{m}{\underset{k=1}{\sum }}R_{k}\cos\left(2\pi f\left(t-t_{\mathrm{meas}}\left(z_{k}\right)\right)\right),$$

where *m* is the number of reflecting surfaces and the *k*-th reflecting surface has the reflectance $R_{k}$ and is placed at distance $z_{k}$ from the 0 point.

The target does not have to be made up of discrete parts; we can consider the reflectance to be a continuous function of the position, R(z), a spatial distribution of the reflectance, with units of reciprocal length. In this case, the sum becomes an integral, as follows:

(12) $$V_{\mathrm{meas}}\left(t\right)=A_{\mathrm{meas}}\int_{-\infty }^{+\infty }R\left(z\right)\cos\left(2\pi f\left(t-t_{\mathrm{meas}}\left(z\right)\right)\right)dz.$$

The purpose of our ranging method is to determine R(z).

Converting $t_{\mathrm{meas}}$ to position gives:

(13) $$t_{\mathrm{meas}}=t_{\mathrm{ref}}+2\frac{z}{c}\;,$$

where c is the speed of light in the medium where the measurement is taken (air). Then the measurement signal becomes:

(14) $$V_{\mathrm{meas}}\left(t\right)=A_{\mathrm{meas}}\int_{-\infty }^{+\infty }R\left(z\right)\cos\left(2\pi ft-2\pi f\left(t_{\mathrm{ref}}+2\frac{z}{c}\right)\right)dz.$$

Then the mixer’s I output can be calculated using Equation (4) again, leading to

(15) $$V_{I}\left(t\right)=A_{\mathrm{ref}}\;A_{\mathrm{meas}}\;g_{\mathrm{mix}}\cos\left(2\pi f\left(t-t_{\mathrm{ref}}\right)\right)\int_{-\infty }^{+\infty }R\left(z\right)\cos\left(2\pi ft-2\pi f\left(t_{\mathrm{ref}}+2\frac{z}{c}\right)\right)dz.$$

Moving the first cosine under the integral and converting the product of cosines into a sum, of which one is again removed by low-pass filtering, we obtain the following simple result:

(16) $$V_{I}=A\int_{-\infty }^{+\infty }R\left(z\right)\cos\left(2\pi f\cdot 2\frac{z}{c}\right)dz,$$

where A is again the resulting amplitude, containing gains and losses in the setup. Similarly, the Q output becomes:

(17) $$V_{Q}=A\int_{-\infty }^{+\infty }R\left(z\right)\sin\left(2\pi f\cdot 2\frac{z}{c}\right)dz.$$

The equations for $V_{I}$ and $V_{Q}$ now look very similar to the real part and the imaginary part of a complex Fourier transform, respectively. We can build the full Fourier transform formula by combining $V_{I}$ and $V_{Q}$ in a complex quantity as follows:

(18) $$V_{I}-iV_{Q}=A\int_{-\infty }^{+\infty }R\left(z\right)\exp\left(-i2\pi f\cdot 2\frac{z}{c}\right)dz,$$

where $i=\sqrt{-1}$.

Equation (18) shows that $V_{I}-iV_{Q}$ is the Fourier transform of R(z). Consequently, R(z) can be calculated from $V_{I}-iV_{Q}$ as its inverse Fourier transform:

(19) $$R\left(z\right)=\frac{2}{Ac}\int_{-\infty }^{+\infty }\left(V_{I}\left(f\right)-iV_{Q}\left(f\right)\right)\exp\left(+i2\pi f\cdot 2\frac{z}{c}\right)df,$$

which was derived from Equation (18) by temporarily changing the integration variable from z to 2z/c; this brings Equation (18) to the canonical form of the Fourier transform and allows a straightforward inversion.

Equation (19) shows that if $V_{I}\left(f\right)$ and $V_{Q}\left(f\right)$ are known at all modulation frequencies, the spatial reflectance distribution R(z) can be calculated exactly.

However, it is impossible to take measurements at an infinite number of modulation frequencies and in an infinite range. In practice, we are necessarily limited in both the range of frequencies and the spacing between consecutive frequencies. The effect of these two limitations is that R(z) cannot be determined exactly; specifically, we will show in the following that both the spatial resolution of R(z) and the range of measurable distances are limited by how we choose the unavoidably discrete frequency values.

### 2.4. Advantage of Measuring Both Cosine and Sine Signals

In the usual SS-OCT technique, only one interference signal is produced, equivalent to our $V_{I}$, such that Equation (19) lacks the imaginary $iV_{Q}$ term. The consequence is that the calculated R(z) becomes symmetrical with respect to z=0, meaning that whether a reflecting surface is placed at position –z or +z, the calculated R(z) will have two peaks, at both –z and +z. In our case, with the addition of the quadrature signal, the negative and positive positions become discriminated and the target position is not confined to just one side of 0.

### 2.5. Resolution

Ideally, the R(z) of a single perfectly reflecting surface placed at position $z_{0}$ is a delta function:

(20) $$R\left(z\right)=\delta \left(z-z_{0}\right).$$

However, when the range of modulation frequencies is limited to the interval from some $f_{\min}$ to some $f_{\max}$, the calculated R(z) will be blurred, because it is convolved by the following function:

(21) $$S\left(z\right)=\int_{f_{min}}^{f_{max}}\exp\left(+i2\pi f\cdot 2\frac{z}{c}\right)df,$$

which is the Fourier transform of a boxcar frequency filter from $f_{\min}$ to $f_{\max}$. This equation calculates to:

(22) $$S\left(z\right)=\frac{\exp\left(+i2\pi 2\frac{z}{c}f_{\max}\right)-\exp\left(-i2\pi 2\frac{z}{c}f_{\min}\right)}{i2\pi 2\frac{z}{c}}.$$

The amount of blurring is determined by the width of the absolute value of S(z), which can be calculated by multiplying S(z) with its complex conjugate and then taking the square root. The result becomes:

(23) $$\left|S\left(z\right)\right|=\left(f_{\max}-f_{\min}\right)\;\mathrm{sinc}\left(\pi 2\frac{z}{c}\left(f_{\max}-f_{\min}\right)\right),$$

where we use the sinc function with the following definition:

(24) $$\mathrm{sinc}\left(x\right)=\frac{\sin\left(x\right)}{x},\;\mathrm{sinc}\left(0\right)=1.$$

The full width at half maximum of the sinc function is approximately π (the two zeros closest to the main peak are at −π and +π). We can now estimate the width of |S(z)|, which we denote by δz:

(25) $$\pi 2\frac{\delta z}{c}\left(f_{\max}-f_{\min}\right)\approx \pi ,$$

(26) $$\delta z\approx \frac{c}{2}\frac{1}{f_{max}-f_{min}}.$$

This is the theoretical resolution of our distance measurement. The conclusion of this subsection is that the calculated R(z) is blurred by a sinc function and that the resolution limit depends only on the available range of modulation frequencies; not much is possible regarding adjusting the speed of light, except for changing the refractive index of the medium, if the application allows. Incidentally, the resolution formula for our DFT radar is identical to that for the FMCW radar.

It should be emphasized here that the resolution is the ability of the radar to resolve the positions of reflecting surfaces that are close to each other in the axial direction, and that it is not the same thing as the distance measurement error. The latter is the error of ranging one reflective surface in the absence of any others, and can be orders of magnitudes smaller.

### 2.6. Distance Measurement Range

In the case of the FMCW radar, the frequency is swept continuously from $f_{\min}$ to $f_{\max}$, but for our DFT radar the frequency is tuned in discrete steps to achieve the homodyne mixing at each step. For that reason, the integral in Equation (19) must be converted into a sum. We chose to change the modulation frequency in n equal steps, from $f_{0}=f_{\min}$ to $f_{n-1}=f_{\max}$:

(27) $$R\left(z\right)\propto \overset{n-1}{\underset{j=0}{\sum }}\left(V_{I}\left(f_{j}\right)-iV_{Q}\left(f_{j}\right)\right)\exp\left(+i2\pi f_{j}\cdot 2\frac{z}{c}\right)\delta f,$$

where δf is the difference between consecutive steps, given by:

(28) $$\delta f=\frac{f_{\max}-f_{\min}}{n-1}.$$

Equation (27) is a discrete Fourier transform, which gives the name to our ranging method.

Since the $V_{I}\left(f_{j}\right)$ and $V_{Q}\left(f_{j}\right)$ datasets are supposed to represent sine waves (for the case of one reflecting surface), in order to satisfy the Nyquist condition, the period of those sine waves must be larger than the sampling step δf by at least of factor of 2, meaning that, in the worst case, we need two data points for each oscillation period. Otherwise, the aliasing effect occurs, in which the data will appear to represent sines with longer periods than the actual ones.

The period of $V_{I}$ (equal to that of $V_{Q}$) can be derived from Equation (7):

(29) $$f_{\mathrm{period}}=\frac{1}{t_{\mathrm{meas}}-t_{\mathrm{ref}}}=\frac{c}{2z}.$$

As the target gets farther away from 0, the period becomes smaller. The Nyquist condition translates into

(30) $$f_{\mathrm{period}}>2\delta f,$$

(31) $$\frac{c}{2z}>2\frac{f_{\max}-f_{\min}}{n-1},$$

(32) $$z<\frac{c\left(n-1\right)}{4\left(f_{\max}-f_{\min}\right)}.$$

This is the maximum distance that can be measured on the positive side; the limit on the negative side is symmetrical. Therefore, the total measurement range ∆z is twice the value above:

(33) $$∆z=\frac{c\left(n-1\right)}{2\left(f_{\max}-f_{\min}\right)}.$$

This formula shows that the measurement range is n−1 times larger than the resolution calculated in Equation (26).

Since Equation (27) is a discrete Fourier transform, for computational reasons it is beneficial if n is chosen to be a power of 2, which allows the use of a fast Fourier transform routine.

## 3. Experimental Verification

The experimental setup shown in Figure 1 has already been explained in the section on the measurement principle. Here we add a few details relevant to the experiments.

We used a sine wave signal generator to modulate the RTD in amplitude at frequencies up to 18 GHz. The power level of the signal generator was adjusted to 8 dBm, which was found to be optimal for the modulation of the RTD output. The IQ mixer needs a higher level in its LO input, in the range 13–18 dBm, so we used an amplifier and an attenuator (not shown in the figure) to adjust the level.

The RTD oscillator used in this experiment is the one described in [5], with an oscillation frequency of 522 GHz and a maximum output power of 10 µW. A hyperhemispherical lens made of high-resistivity silicon is attached to the RTD chip and transmits the terahertz wave to the free space while also reducing the initial 180° beam divergence angle to a more manageable few tens of degrees.

For our radar, the ability to modulate the amplitude of the RTD oscillator is critical. This is achieved by setting the RTD bias voltage at a point where the output power is about half of the maximum (0.56 V for this particular device), and then superimposing the modulation signal on that bias voltage. The modulation signal is usually sinusoidal, but the nonlinearity of the power–voltage characteristic means that the modulated signal will also unavoidably contain harmonics of the modulation frequency; we have not noticed any effect of these harmonics on the measurements. The modulation frequency of an RTD can be as high as 30 GHz for specifically fabricated chips intended for use in communications [15]. The chip we used here has a −3 dB cutoff frequency below 10 GHz; the frequency-dependent amplitude could be compensated at least in part by using stronger modulation signals at higher frequencies, but, for the sake of simplicity, we used a constant level.

The terahertz beam emitted by the RTD is collimated using a plastic lens such that the parallel beam has a diameter of about 47 mm. The beamsplitter, the mirrors used as targets, and the second lens are sufficiently wide so as not to clip the beam. The total propagation length in air is just below 1 m; in these conditions, we do not expect diffraction to be a problem.

The beamsplitter is custom-made from high-resistivity silicon with a thickness around 0.3 mm, selected such that the transmission and the reflection coefficients are both around 50% at the 45° incidence angle and the particular polarization orientation of our RTD. We used a flat gold-coated glass mirror as target when a full mirror was needed. When testing the system on two targets, we used this full mirror together with a half mirror placed in front of it; for the half mirror, we used another high-resistivity silicon plate, similar to the beamsplitter. The full mirror is set on a motor stage that can move over a distance of 200 mm with a specified positioning repeatability of 15 µm.

The detector is a Fermi-level-managed barrier diode (FMBD) with a confirmed sensitivity from 200 GHz to 1 THz [16]. The FMBD module includes a transimpedance amplifier that allows modulation frequencies up to about 11 GHz (at −3 dB). Two additional external low-noise amplifiers further boost the signal. We found in another experiment that the signal-to-noise level of the FMBD detector is somewhat better than that of a commercial Schottky-barrier-diode (SBD) detector, up to 10 dB depending also on the modulation frequency, so we preferred the FMBD for this experiment.

The two outputs of the IQ mixer are connected to two low-frequency amplifiers that feature adjustable gain and adjustable low-pass filters. The signals are then digitized and sent to a computer. The same computer controls the signal generator and the motor stage. A LabVIEW program sends commands to the signal generator to adjust the modulation frequency, reads the detected signals, and processes them.

### 3.1. Measurement Results on a Fixed Target

**Figure 2:**
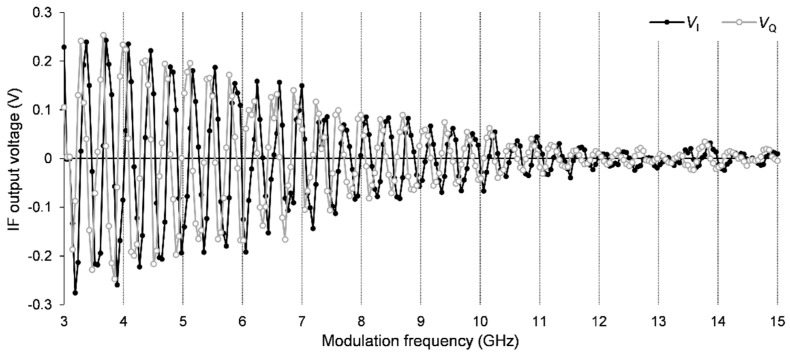
Measured IF output signals in the case of a single target, when the motor stage is placed at 0. The modulation frequency was scanned from 3 to 15 GHz in 256 steps.

Figure 2 shows the measured $V_{I}$ and $V_{Q}$ signals for the case when the target is a full mirror placed at a fixed position. The modulation frequency was scanned from 3 GHz to 15 GHz in 256 steps. The graph shows that the two signals are periodic in frequency, with a quarter-period shift between them. The amplitude of the voltage variation is not constant; at higher frequencies, the amplitude decreases mainly due to the RTD internal circuit, which contains an RLC network manifesting itself as a low-pass filter.

**Figure 3:**
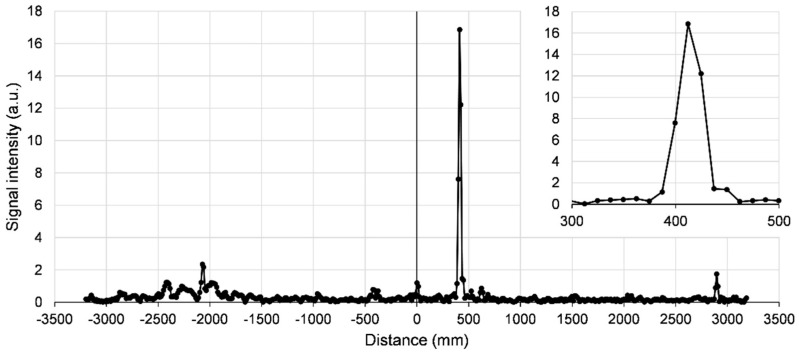
The inverse Fourier transform of the signals in Figure 2. The horizontal axis is converted into distance; the vertical axis is the absolute value of the complex number produced by the Fourier transform. The inset is an expansion around the main peak and shows that the distance information is mainly contained in just three data points.

Figure 3 shows the inverse Fourier transform of the $V_{I}-iV_{Q}$ quantity, which reveals the absolute distance to the target by the position of the main peak. Smaller peaks are also visible; they are most probably caused by reflections inside cables by imperfect connections or by reflections in the optical part of the setup. These are not easy to remove, but could be avoided by lengthening or shortening the cables and/or the propagation paths in air, so that the range of interest is relatively free from such peaks.

We have not calibrated and corrected the IQ mixer outputs in any way. We know from other experiments and from the mixer’s datasheet that the I and Q outputs suffer from three particular imperfections: first, there are voltage offsets present in the two outputs even in the absence of any RF input; second, the variation amplitudes of the two outputs are not exactly equal; and third, the phase shift between them is not exactly 90°. The effects of these imperfections can in fact be seen in Figure 3: the small peak at 0 is likely caused by voltage offsets, while the small peak at around −400 mm (symmetrical of the main peak at +400 mm) could be an effect of the unbalanced amplitudes and the inexact phase shift. In our proof-of-concept experiments, we have not attempted removing these peaks, but in principle this could be achieved through calibration and correction.

To improve the accuracy of the distance measurement, the arrays of measured $V_{I}$ and $V_{Q}$ values are first multiplied by an apodization window function before passing them through the inverse Fourier transformation. The position of the resulting peak in the Fourier transform is then determined by fitting the data with a peak function. The precision of distance measurement depends directly on which combination we use for the window function and the fitting function. Poorly performing combinations lead to large, periodic, systematic errors, when the target is moved linearly.

We tested several combinations and chose a truncated Gaussian for the window function and another Gaussian for the peak fitting. While not ideal, this pairing turned out to provide the smallest systematic errors. The amount of Gaussian truncation in the window function was optimized so as to minimize the errors. If the Gaussian window function is too wide, the apodization effect is too weak. On the other hand, if the Gaussian window is too narrow, then too much of the measured data is discarded and the noise affects the error; in addition, this also weakens the resolution. We believe it should be possible to remove the systematic error entirely by determining what peak-fitting function matches the peak shape precisely. To determine the peak shape, a Fourier analysis of the envelope of the signals in Figure 2 would be necessary; we have not attempted this so far.

### 3.2. Measurement Results on a Movable Target

The previous section describes the signals obtained on a single fixed target. However, to estimate the accuracy of our ranging method, we would need to know the actual distance to the target in absolute terms. In practice, we are not able to perform such an independent measurement with sufficient accuracy, since it would require measuring all propagation times with an accuracy much better than that which our DFT radar can provide.

We can, however, measure the precision, which is the statistical variability of the measurement results, excluding the absolute part. For that purpose, we moved the target to a series of precisely known positions and used the DFT radar to measure the distance for each of them.

**Figure 4:**
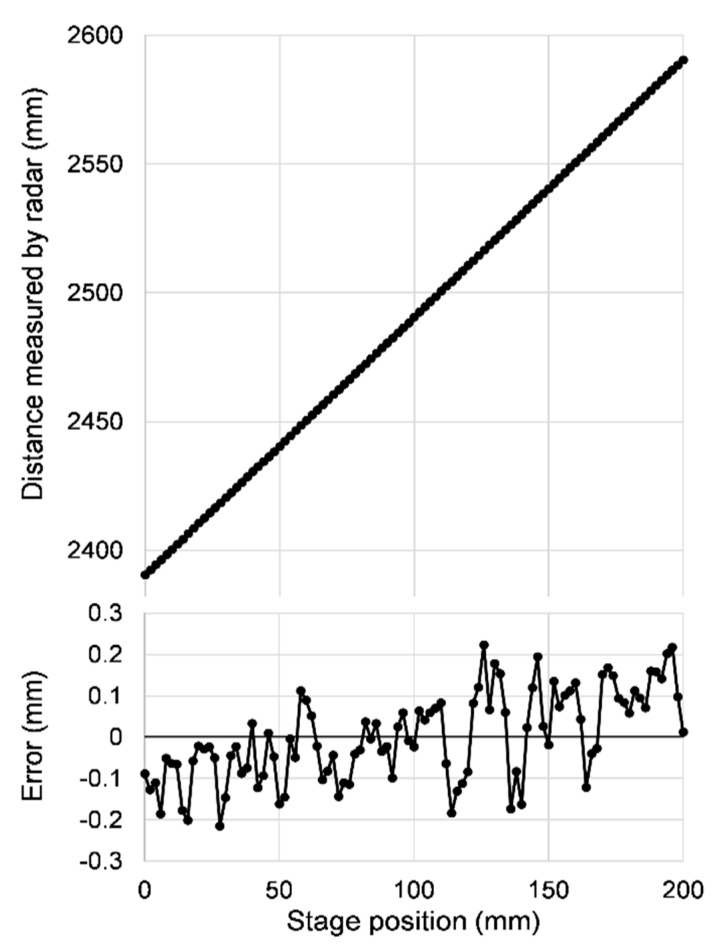
Distance measurement on one target at various positions of the motor stage, in 2 mm steps. The modulation frequency was scanned from 3 to 15 GHz in 512 steps. The top plot shows the absolute distance measured by the radar versus the stage position. As the measurement errors are too small to see at this scale, the bottom plot is added to show just the error, defined as the difference between the measured distance at each point and a linear fit through all points, with the slope fixed to 1. The standard deviation of the errors is 0.107 mm.

Figure 4 shows the result of such an experiment, in which a flat gold-coated glass mirror was used as target, placed on the motor stage. The upper plot shows how the distance measured by the radar depends linearly on the relative position of the motor stage carrying the target. No departure from linearity can be seen at this scale. We fit the measured data to a linear function of slope fixed to 1 and calculated the measurement error of each point as the difference between the measured distance and the linear fit. The result is shown in the lower graph.

The standard deviation of the errors turns out to be 0.107 mm. This includes the effects of noise and any residual systematic errors caused by the peak fitting. The points in the error plot appear to have a slight upward slope, but this is accidental, just the effect of fluctuations. Every time we repeat the measurement the apparent slope changes randomly in size and in sign.

We can assume that the accuracy—the closeness of the measured distances to their actual absolute values—is at the same level as the precision, since at least theoretically there is no particular reason for a systematic constant shift affecting the calculated distance.

### 3.3. Distance Measurement on Two Targets

In order to check the ability of the DFT radar in measuring more than one distance at once, and also to estimate the ranging resolution, we also made measurements on two targets. The terahertz beam is first partially reflected and partially transmitted by the half mirror, then the transmitted part is reflected back by the full mirror. Repeated reflections between the two targets result in signals that are too weak to be detected. The full mirror was moved over a 200 mm range; when the motor stage is at its home (0 mm) position, the distance between the targets is the shortest, about 10 mm.

In addition, in some of the measurements we also placed a piece of fabric (a regular cotton handkerchief) over the fixed half mirror, such that the fabric covered both of its sides. In total, the terahertz beam passes through the fabric twice for the half mirror and four times for the full mirror. As the fabric is not held flat and perpendicular to the beam, it does not create its own peaks in the Fourier transform.

**Figure 5:**
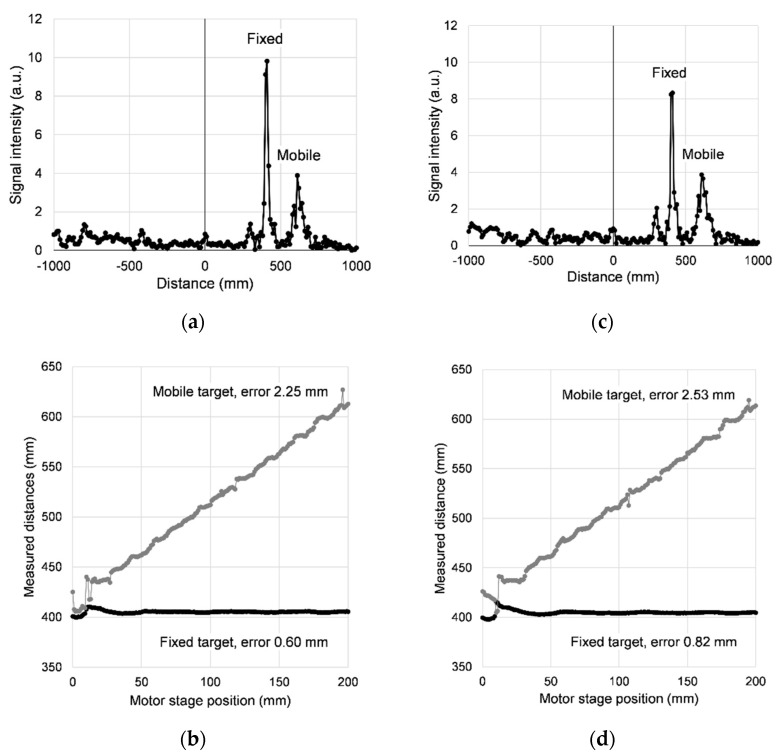
Measurement results on two targets, a fixed half mirror and a mobile full mirror; the modulation frequency had 512 values from 3 to 18 GHz. (**a**) The Fourier transform when the mobile target is in its most distant position from the fixed target, with the motor stage at 200 mm. (**b**) The distance to the two targets as measured by the DFT radar; the ranging errors included in the labels exclude the first 20 mm part of the motor stage position, where the two peaks are not clearly resolved. (**c**,**d**) The corresponding data for the case where a layer of cotton fabric was inserted in the beam on each side of the half mirror. The measurement errors are slightly larger.

Figure 5 shows the results of these experiments. The two peaks corresponding to the two targets are sufficiently well resolved to allow individual ranging, with the exception of the first 15 mm or so of the motor stage position, where the targets are too close to each other. We used a very basic procedure for detecting the peaks and no attempt was made towards better signal processing, such as multipeak fitting.

To evaluate the measurement precision in Figure 5b,d, the same linear fitting was used as in the case of a single target, but obviously the slope of the fixed target was set to 0; for the mobile target the slope was set to 1. In addition, the leftmost part of the data was excluded, as the peak detection in that range was unreliable, and only the range from 20 to 200 mm of the horizontal axis was used. With these provisions, the standard deviation for the half mirror is found to be 0.60 mm, whereas for the full mirror it is 2.25 mm. When the fabric is inserted into the beam, both errors increase slightly, to 0.82 and 2.53 mm, respectively, which can be attributed to a degraded signal-to-noise ratio.

By extrapolating the two lines to the left and finding the intersection point, we can also calculate the distance between the targets when the motor stage is at 0. This offset distance is found to be about 10 mm, both without and with the fabric, which matches the actual distance in the optical setup with an error of less than 1 mm.

Considering that the fixed mirror is offset by 10 mm away from the mobile mirror, and that the peaks start being resolved reliably when the motor stage is at 15 mm, the physical distance between the targets at this point is 25 mm. This means that the ranging resolution of our radar is roughly 25 mm. The theoretical resolution, as given by Equation (26), should be 10 mm at our bandwidth of 15 GHz.

The mismatch between theory and experiment can be explained by two factors. Firstly, in the case of the RTD source, the amplitude of the signal at higher modulation frequencies is very small, such that only the lower frequencies contribute significantly, whereas the theory assumes a flat frequency response. Secondly, the apodization window further reduces the contribution of the lowest modulation frequencies. The result is that the effective bandwidth is considerably narrower than the 15 GHz that we plugged into the theoretical formula.

## 4. Discussion

Compared with our implementation of the AMCW radar based on an RTD source [5,6], the DFT radar has the great advantage of being able to measure more than one distance at a time, despite being only marginally more complex. On the other hand, compared with our RTD-based FMCW radar [4], we found that the DFT principle could be beneficial in practical situations, particularly because it does not require a frequency sweep and produces only constant voltage IF signals that are easy to measure and process, although otherwise it provides the same ranging resolution.

By combining the DFT radar with one- or two-dimensional beam scanning, a possible application could be found in cross-sectional imaging.

As the RTD output powers and available oscillation frequencies progressively improve with ongoing research, we anticipate that the range of applications will widen, in a way that might parallel the history of devices such as the light-emitting diode. Currently, RTD oscillation frequencies up to 1.98 THz are demonstrated [17], although simulations show it could reach even higher, to about 3 THz [18]. The output power is now in the microwatt order, but an incoherent array of RTDs was shown to emit 0.73 mW [19] and research is ongoing toward a single-element power emission of several milliwatts [20,21]. We expect that these improvements, together with the benefit of a very small device size and potentially a reduced cost, will increasingly cause users to prefer RTD sources in applications.

## 5. Conclusions

We described a distance measurement principle that we call DFT radar, and presented its experimental verification for the particular case where we used terahertz waves emitted by an RTD oscillator and detected by an FMBD sensor. The principle of this radar places it somewhere between the AMCW radar and the FMCW radar; with the former, it shares the use of fixed modulation frequencies; as with the latter, it features a multi-target detection capability.

In this proof-of-concept implementation, where performance optimization was not a priority, we found that we can measure distances with a precision of 0.107 mm on a single target. On two targets, the signals become weaker and the precision worsens, but this experiment confirmed that it is possible to detect at least two targets simultaneously. To showcase the benefit of using terahertz waves, in another experiment we proved that the precision is only slightly affected by passing the beam through layers of fabric. The radar’s ability to resolve targets axially was found to be roughly 25 mm, which is worse than the theoretical 10 mm due mainly to a decrease in signal levels at higher modulation frequencies.

Improvements can be expected from the future availability of RTD oscillators with higher output powers and better modulation properties.
